# Duration of Untreated Illness in Patients with Obsessive–Compulsive Disorder and Its Impact on Long-Term Outcome: A Systematic Review

**DOI:** 10.3390/jpm13101453

**Published:** 2023-09-29

**Authors:** Francesco Perris, Salvatore Cipolla, Pierluigi Catapano, Gaia Sampogna, Mario Luciano, Vincenzo Giallonardo, Valeria Del Vecchio, Michele Fabrazzo, Andrea Fiorillo, Francesco Catapano

**Affiliations:** Department of Psychiatry, University of Campania “L. Vanvitelli”, 80138 Naples, Italy

**Keywords:** obsessive–compulsive disorder, duration of untreated illness, long-term outcomes, prognosis, early intervention

## Abstract

***Background***: Duration of untreated illness (DUI)—defined as the time period between the onset of a mental disorder and its first adequate treatment—should influence patients’ long-term prognosis and outcome. In patients with obsessive–compulsive disorder (OCD), DUI lasts on average from 87.5 up to 94.5 months, being significantly longer compared with data available from patients affected by other severe mental disorders, such as schizophrenia and bipolar disorder. We carried out a systematic review in order to assess the impact of DUI on long-term outcomes in OCD patients. ***Methods***: A systemic review has been implemented, searching from inception to April 2023; only papers written in English were included. ***Results***: Seventy-one articles were initially identified; only eight papers were included in the review. The DUI ranged from 7.0 ± 8.5 to 20.9 ± 11.2 years. Patients reporting a longer DUI have a poor long-term outcome in terms of lower level of treatment response and greater symptom severity. ***Conclusions***: The present review confirms that longer DUI has a negative impact on the long-term outcome of patients with OCD. It should be useful to promote the dissemination of early interventions with a specific focus on OCD symptoms.

## 1. Background

Obsessive–compulsive disorder (OCD) is a clinical condition characterized by the presence of obsessions and compulsions, but its clinical onset and presentation can greatly vary [1,2]. The lifetime prevalence is about 2.3% in the general population [3], with two peaks of incidence at 11 and 23 years [4].

Duration of untreated illness (DUI) [5]—defined as the period between the onset of a mental disorder and its first adequate treatment—has been investigated as a potential modifiable risk factor for patients’ long-term outcomes. The relationship between DUI and outcome was originally found in people affected by schizophrenia spectrum disorders [6,7,8]. Based on those studies, the paradigm of care of early detection and early interventions for people with psychosis was initially developed [9]. Nowadays, the paradigm of early interventions in psychiatry represents a relevant model of care for treating people with severe mental disorders. In fact, the long-term trajectory of any mental disorder is influenced by treatments provided in the first years following the onset of the disease [1,10,11]. Moreover, the conceptualization of mental disorders is shifting from a categorical approach to a dimensional and transnosographic one [12,13,14], which further highlights the importance of the early detection of “any” signs or symptoms of mental distress, which could evolve into a specific full-blown condition. In order to design and disseminate innovative models of care for people suffering from mental disorders such as OCD, schizophrenia, or affective disorders, it is necessary to have clear data on the possible negative impact of DUI on long-term outcomes. However, the relationship between DUI in schizophrenia and outcome is far from being resolved, as recently pointed out by Moritz et al. [15], which highlighted that data are controversial and its applicability to those who are considered at risk remains elusive. To date, few studies have been focused on the impact of DUI on long-term outcomes in people suffering from other mental disorders, including anxiety disorders and obsessive–compulsive disorder (OCD) [16,17]. From the few available studies, in patients with OCD, DUI lasts from 87.5 up to 94.5 months, on average, being significantly longer compared with data available from patient population affected by other severe mental disorders [18,19,20]. Longer DUIs can lead to brain structure alterations and have been reported to cause cortical thinning in the right hemisphere [21], leading to reduced responses to pharmacological treatment [17]. A reduction in the DUI may lead to better treatment outcomes, resulting in earlier improvement in quality of life (QOL). To reduce the DUI, earlier access to a psychiatrist for a patient must be facilitated and dropping out of treatment must be prevented.

DUI usually represents 40–70% of global illness duration, especially when onset occurs during childhood and/or adolescence and the first correct diagnosis and adequate treatment occur in adulthood. Several factors contribute to longer DUI in OCD patients, including the insidious onset of obsessive–compulsive symptoms, the delay in help-seeking due to the misconception regarding the self-limiting course of obsessive symptomatology, stigma [21], and the lack of dedicated mental health care services for early diagnosis in OCD [18,22,23,24,25,26].

We carried out a systematic review of the available literature in order to clarify the impact of DUI on the long-term outcome of people with OCD. Although patients and clinicians often prioritize different outcomes for defining the concept of “remission”, for the scope of the present study, “remission” has been defined as a Yale–Brown Obsessive–Compulsive Scale (YBOCS) score of ≤12, which has been considered a good cut-off to predict a clinical state where, if residual symptoms are present, they do not interfere with everyday life [27].

## 2. Materials and Methods

A literature search on PubMed, APA PsycInfo, and Scopus databases has been performed, entering the following keywords: “obsessive-compulsive disorder”, “OCD”, “duration of untreated illness”, and “DUI”. The search method was conducted according to the Preferred Reporting Items for Systematic Review and Meta-Analysis (PRISMA) statement, as applicable [28]. The search was carried out from the earliest available date in each respective database to April 2023, and only papers written in English were included. The reference lists of included articles were screened for identifying additional relevant studies.

The following inclusion criteria were used: (1) studies reporting DUI in a sample of adult OCD patients; (2) studies using rating scales for the assessment of severity of clinical symptoms and levels of psychosocial functioning; (3) studies focused on DUI and its impact on outcome in patients with OCD; (4) studies containing data on the differences in treatment response measured by standardized rating scales between patients with different DUI as primary outcome. If the study sample is composed of people affected by more than one comorbid psychiatric disorder, OCD had to be the primary diagnosis, defined as the disorder causing the most significant distress and alteration of functioning, representing the primary reason to seek treatment. Articles have been screened, selected, and extracted by two authors (FP and SC); two other authors (GS and MF) checked the accuracy of the extracted data. Two authors (AF and FC) independently assessed the quality and the risk of bias in the non-randomized studies of interventions (NRSIs) included in the review using the ROBINS-I tool (Risk of Bias in Non-randomized Studies of Interventions) [29], which includes three main domains for bias evaluation: pre-intervention, during intervention, and post-intervention. The risk of bias was judged for each domain and sub-domain and classified as low, moderate, high, or no information (Supplementary Table S1). In case of disagreement, a senior author was included in the discussion (FC). Main information of selected studies, including author, study design, sample size, methods, outcome assessment, mean DUI (expressed in years), and main findings, have been extracted. Considering the low number of papers included in the review and the heterogeneity in evaluating DUI and its impact on long-term outcomes, it was not possible to perform a meta-analysis.

## 3. Results

**Figure 1:**
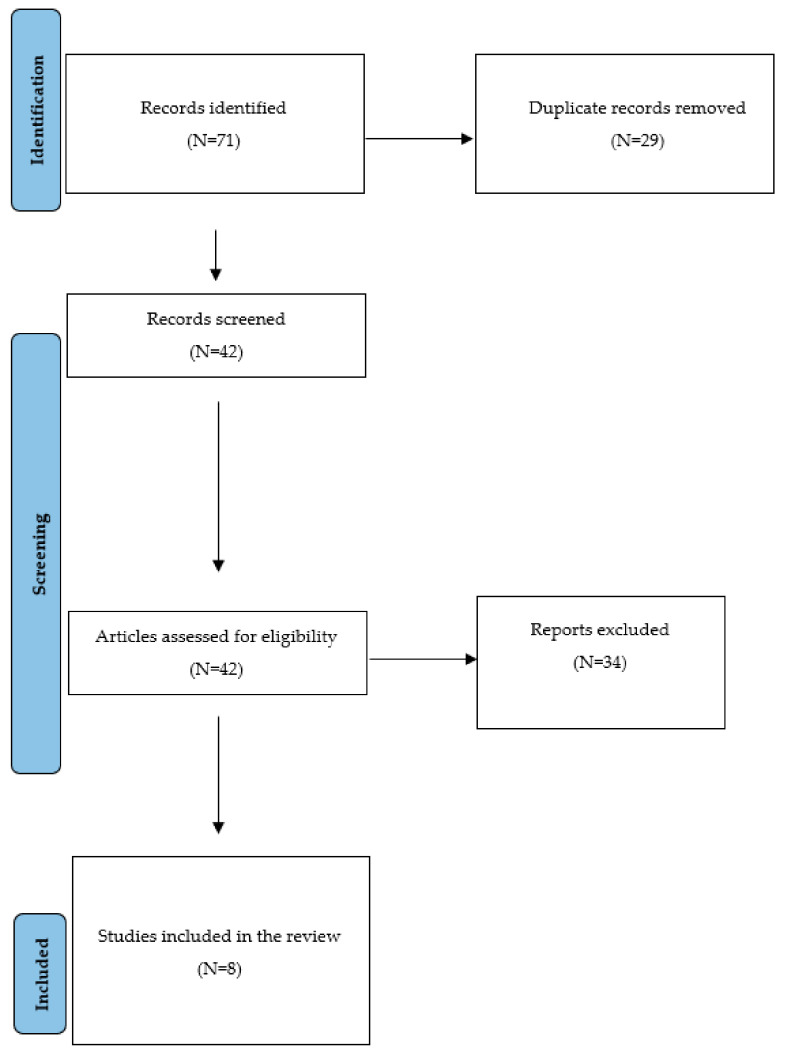
PRISMA flow diagram of selection of studies for inclusion in the review.

The selection process is summarized in Figure 1. Seventy-one articles were initially identified; of these, twenty-nine papers were removed as duplicates; therefore, 42 paper were screened using the title and abstracts and N = 34 papers were excluded as not addressing the topic of interest, and eight articles were finally included. The overall risk of bias was moderate for most selected studies (5/8), low for one study, and high for two studies (Supplementary Table S1).

The main findings are summarized in Table 1. A longitudinal study design was adopted in four studies, while the remaining were cross-sectional (N = 3) and retrospective (N = 1). Only one study included adolescent patients [29]. In all included studies, the Structured Clinical Interview for DSM-IV Axis I Disorders (SCID-I) has been used to confirm the diagnosis.

Different validated and reliable assessment tools have been used, including the Yale–Brown Obsessive–Compulsive Scale (Y-BOCS) (used in all studies), the Hamilton Anxiety Rating Scale (HAM-A) and the Hamilton Depression Rating Scale (HAM-D) [17], the Clinical Global Impression (CGI) [37], the Hamilton Depression Rating Scale (HDRS), the Brown Assessment of Belief Scale (BABS) [34], and the Global Assessment of Functioning (GAF) [35]. In the study by Poyraz et al., an ad hoc questionnaire consisting of 16 items was used in order to assess the reasons for treatment delay [33].

Mean DUI ranged from 7.0 (±8.5) years in the Poyraz study [33] to 20.9 (±11.2) years in the Jakubovski study [31].

Dell’Osso et al. found that DUI, considered as a continuous variable, does not predict treatment response or remission [30]. When considered a categorical variable, a DUI ≥ 24 months is predictive of poor treatment response. Jakubovski et al. found a better prognosis in patients with late-onset OCD and a poorer outcome in OCD patients with comorbid affective disorders [31]. Dell’Osso et al. found a mean DUI of 87.35 ± 11.75 months (approximately 7 years), which varies according to different clinical subtypes of OCD. In particular, patients reporting aggressive/checking symptoms have longer DUI and DI, which may be due to a lack of insight and reluctance toward help-seeking in this patient population [32].

Moreover, the long-term outcome and remission in patients with OCD can be negatively influenced by the presence of comorbid psychiatric conditions [37]. In this study, patients with a severe type of disease were young, with a high lifetime rate of psychiatric comorbidity, very early onset OCD, long DI and most notably, a shorter DUI. This could be due to the fact that a more severe clinical presentation can lead to earlier treatment seeking, with a shorter DUI.

A positive response to pharmacological treatment—evaluated as a reduction in terms of the YBOCS scale—is significantly lower in patients with a DUI longer than 24 months, with a response rate of 41% vs. 69% [17].

In an ongoing longitudinal real-world study carried out by Perris et al., several factors are associated with a long DUI, including unemployment, early onset and more severe symptoms at baseline, with a strong statistical correlation between DUI and outcome [35]. Finally, Zheng et al. found a higher response rate in patients with a shorter DUI compared to those with a longer DUI, confirming the existence of a DUI-dependent effect on post-treatment outcome [36]. Only one cross-sectional study found no effect of DUI on long-term remission [33].

## 4. Discussion

The duration of untreated illness represents a critical element for the long-term prognosis of OCD patients. The negative impact of DUI on the long-term outcomes of OCD patients is confirmed in the present systematic review.

In particular, we found that patients with a longer DUI have a higher risk of reporting inadequate treatment response, persistence of severe symptoms, and low rate of remission. This is particularly true when the onset of the disease is insidious and subthreshold. As suggested by Dell’Osso et al., the predictive effect of DUI on treatment response may vanish after a certain period of time since its negative effect occurs mainly in the early years of the disease [30].

Positive response to pharmacological treatment is significantly reduced when patients have a long DUI [17], in line with data coming from a sample of patients suffering from psychosis or schizophrenia spectrum disorders [31,33].

Therefore, these findings confirm the need for designing and scaling up effective and innovative interventions specifically focused on early detection and management of OCD patients, following the same model of care developed for people with psychosis [20].

Multicomponent and multilevel interventions should include informative campaigns for young people to be disseminated in schools, the promotion of antistigma campaigns through the use of social media on the importance of early referral to specialistic care and help-seeking [38], and the establishment of non-stigmatizing mental health care facilities dedicated to young people needs, in order to facilitate access and appropriate mental health care [39,40,41,42,43,44,45,46].

Moreover, DUI can play a significant role as a long-term predictor of response to any pharmacological and non-pharmacological treatment. As reported in Table 1, different pharmacological (including fluoxetine, venlafaxine, clomipramine or combined treatments) and non-pharmacological treatments (such as CBT) have been used. However, different treatments have been provided according to the available guidelines for treating patients with OCD. Therefore, the type of treatment should not have a specific impact on DUI, but it was out of the scope of the present review to specifically assess this aspect. However, in order to personalize the treatment plan for patients with OCD, it should be useful to combine DUI with sociodemographic, psychosocial and clinical data in machine learning approaches for predicting patient outcomes [47,48,49,50,51].

Our systematic review has some limitations that should be acknowledged. First, the search strategy has been limited only to studies including adult patients aged over 18 years. This methodological choice was due to the fact that the presentation of OCD in late childhood and/or adolescence can have different clinical and psychosocial characteristics, which are usually assessed through specific assessment tools specifically validated for young populations. Therefore, a further literature search with a specific focus on patients with a childhood/adolescent onset of OCD should be performed, and the results could be useful to support the development of youth mental health services [46,52,53].

Moreover, in all studies, DUI has been assessed through interviews including the patients but also family members, caregivers or referring clinicians, which may have yielded inaccurate reports. However, this is a limitation common to all studies focusing on DUI, which is usually retrospectively described and, therefore, subjected to recall bias. Another limitation is the heterogeneity of studies; in particular, the symptomatic phenotype, the comorbidities with personality disorders and patients’ insight were not investigated by Zheng et al. [36]; Perris et al. [35] found a very high dropout rate (about 28%). Socio-cultural factors, including religion and personal beliefs, can influence the clinical manifestations of symptomatology as well as the help-seeking delay and the type of professionals contacted. Therefore, all these variables can impact the duration of untreated illness. All these aspects have not been specifically evaluated in the present review since those data have not been specifically reported in the included studies. Furthermore, no study has specifically evaluated the impact of the COVID-19 pandemic on the duration of illness and help-seeking delay [11,54].

The limited number of included studies (only 8 out of the 42 identified) highlights that the topic of duration of untreated illness has been overlooked in patients with OCD. Our results should be further confirmed by rigorous longitudinal cohort studies aiming to assess the relationship between the duration of untreated illness and OCD patients’ long-term outcomes. Another controversial issue is related to the management of DUI as categorical or continuous variables. There is no consensus, and only a study by Dell’Osso et al. [30] considered DUI both as a categorical and continuous variable, highlighting some differences. Further studies should be promoted in order to clarify this issue.

## 5. Conclusions

Following studies on the role of the duration of untreated illness in patients with psychosis, a care model based on early intervention services was developed worldwide. In fact, it has been repeatedly confirmed that the long-term trajectory of any mental disorder is highly influenced by treatments provided to patients in the first years of the disease [54,55,56]. Therefore, the early detection and appropriate treatment of any mental disorder is essential to improve the long-term prognosis of patients [57,58,59,60,61,62]. This model must also be applied to other severe mental disorders, including obsessive–compulsive disorder, which has been overlooked and wrongly considered “less” severe compared to psychosis. A possible negative impact of DUI on the long-term outcomes of patients with OCD has been highlighted by the present systematic review. However, considering the limited number of studies identified and the presence of some methodological limitations, a definitive conclusion cannot be made. It is necessary to promote more rigorous research studies in order to clarify the potential role of DUI on the long-term outcomes in patients with OCD.

## Figures and Tables

**Table 1:** Summary of data extracted from selected studies and short evaluation of the correlation between longer DUI and worse outcomes.

First Author (Publication Year)	Type of Study	Sample	Methods	Outcome Assessment	Mean DUI (Years)	Results	Longer DUI Worse Outcome
Dell’Osso et al. (2010) [30]	Longitudinal study	66 patients with primary diagnosis of OCD according to DSM-IV-TR. Two groups: DUI ≤ 24 months and DUI > 24 months. Two subgroups: monotherapy and polytherapy.	SCID-I and SCID-II at baseline for clinical assessment. Y-BOCS at baseline and after 12 weeks of pharmacological treatment to measure outcome.	Response to treatment: Y-BOCS decrease > 25% Remission: Y-BOCS score ≤ 10	7.75 * (±9.24)	DUI considered as a continuous variable does not influence treatment response. DUI ≤ 24 months is predictive of treatment response (OR = 0.27; *p* = 0.03) but not of remission (OR = 0.41; *p* = 0.12). It suggests the existence of a time-dependent effect of the DUI, that, after a certain period of time, may vanish.	YES
		Anti-obsessive treatment consisted of monotherapy (SSRI) or polytherapy (combination of SSRI with benzodiazepines, mood stabilizers, antipsychotics, or clomipramine).					
Jakubovski et al. (2013) [31]	Longitudinal study	196 patients with primary diagnosis of OCD according to DSM-IV (only 75 continued 2 years of follow-up). Two groups: SSRI (n = 108) and GCBT (n = 88) treatment.	SCID-I and Y-BOCS at baseline for clinical assessment. Y-BOCS, BDI, BAI at baseline and after 3,6, 12, 18 and 24 months.	Response to treatment: Y-BOCS decrease > 35% Remission: Y-BOCS score ≤ 8	20.87 (±11.25)	Patients who suffered from OCD for a period of 30 years or longer had consistently higher Y-BOCS scores and did not further improve over time. Early onset of symptoms and longer duration of illness seem interconnected.	YES
		Patients allocated to pharmacological treatment received fluoxetine up to 80 mg/day. Patients allocated to GCBT attended 12 weekly therapy sessions. Subsequent treatment options for non-responders were: CGBT + SSRI; switching SSRI; SSRI + clomipramine; SSRI + quetiapine/risperidone; combination of pharmacologic add-on therapy + CGBT					
Dell’Osso et al. (2015) [32]	Cross-sectional study	114 patients with primary diagnosis of OCD according to DSM-IV-TR. Four subgroups based on clinical phenotypes: checking/aggressive, contamination/cleaning, symmetry/order, and multiple phenotypes.	SCID-I for clinical assessment; Y-BOCS to define OCD severity; Y-BOCS Symptom Checklist to identify clinical phenotypes.	Y-BOCS scores	7.27 * (±0.97)	DUI and DI were significantly higher in the aggressive/checking subgroup compared to the other subgroups (*p* < 0.01). Y-BOCS scores were significantly higher in the aggressive/checking subgroup. This result may indicate a greater severity for this phenotype, but it may also be related to longer DUI and DI per se.	YES
		All patients were on a stable pharmacological treatment for at least 4 weeks.					
Poyraz et al. (2015) [33]	Cross-sectional study	96 patients with primary diagnosis of OCD according to DSM-IV-TR. Two groups: DUI ≤ 4 years and DUI > 4 years.	SCID-I and SCID-II for clinical assessment; Y-BOCS to define OCD severity; Y-BOCS Symptom Checklist; a questionnaire to identify reasons for delaying treatment.	Remission: Y-BOCS score ≤ 10	7.02 (±8.52)	Patients with early onset (<12 years) of symptoms had a significantly longer DUI (*p* = 0.001). DUI was not predictive of remission when DUI was considered as a continuous variable or as categorical variable. In logistic regression, DUI was not predictive of remission (OR = 1.1; *p* = 0.074), but *p*-values indicated a distinct trend toward significance.	NO
		50 patients were on SSRIs and/or clomipramine, 44 patients were on different augmentation strategies including SSRIs and/or clomipramine and antipsychotic mood stabilizers.					
Dell’Osso et al. (2017) [34]	Cross-sectional study	124 patients with primary diagnosis of OCD according to DSM-5. Two groups: Y-BOCS score ≤ 24 and Y-BOCS score > 24	SCID-I and SCID-II for clinical assessment; Y-BOCS to define OCD severity; Y-BOCS Symptom Checklist to identify clinical phenotypes; GCI score.	Y-BOCS scores	7.29 * (±9.06)	The group with increased severity received first pharmacological treatment earlier than the other group, consequently reporting a shorter DUI (*p* < 0.01). This could possibly be due to a worse clinical presentation leading to an earlier seeking of treatment.	NO
		Pharmacological treatment based on antidepressant drugs.					
Albert et al. (2019) [17]	Retrospective study	251 patients with primary diagnosis of OCD according to DSM-IV (only 240 had a baseline and a 12-week Y-BOCS to determine response rate). Two groups: brief DUI (≤24 months) and long DUI (>24 months). Two different groups: DUI below median (≤60 months) and DUI above median (>60 months)	SCID-I and SCID-II for clinical assessment. OCD severity assessed by Y-BOCS, Y-BOCS Checklist, HAM-A, HAM-D.	Response to treatment: Y-BOCS decrease ≥ 25%	8.84 * (±9.84)	Long DUI (>24 months) reduces response rates (41% vs. 69%) as well as above the median DUI (>60 months) (40% vs. 61%). Mean DUI is significantly longer in subjects not responding to the first adequate SRI treatment. In individuals with long/above median DUI, Y-BOCS scores at 12 weeks were higher and percentage changes in Y-BOCS scores lower. In regression analyses, DUI > 24 months predicted response and 12-week Y-BOCS scores, but not using DUI as a continuous variable.	YES
		All patients treated with clomipramine and/or SSRIs for at least 12 weeks at adequate doses.					
Perris et al. (2021) [35]	Longitudinal study	83 patients with primary diagnosis of OCD according to DSM-IV (59 completed 3 years follow-up).	SCID-I, SCID-II and BABS at baseline for clinical assessment. Y-BOCS and HADRS administered at baseline and monthly (for the first year of follow-up) or every two months (for the remaining 2 years of follow-up).	Response to treatment: Y-BOCS decrease > 35%. Partial remission: Y-BOCS < 15 for at least 8 weeks. Full remission: Y-BOCS < 8 for at least 8 weeks.	7.3 (±5.8)	Patients with “good outcome” (defined as fulfilling criteria for partial remission for more than 40% of the follow-up period) showed a shorter DUI than patients with “poor outcome” (4.5 ± 3.1 years versus 10.1 ± 5.7 years; *p* < 0.001). In the logistic multivariable model, a short DUI was the only significant predictor of “good outcome”.	YES
		First-line treatment: 25 individual ERP session + SSRI. Add-on strategy in resistant patients: venlafaxine; mirtazapine; imipramine. Second-line treatment: low dosages of antipsychotics as add-on therapy. Benzodiazepines to manage sleep disorder and/or panic attacks.					
Zheng et al. (2021) [36]	Longitudinal study	207 patients with primary diagnosis of OCD according to DSM-5. Two groups: DUI ≤ 3 years and DUI > 3 years.	SCID-I at baseline for clinical assessment. GAF at baseline to evaluate overall functional impairment in the past month. Y-BOCS at baseline and after 8, 12, 24, and 48 weeks of pharmacological treatment to measure outcome.	Partial response: Y-BOCS decrease > 25%. Full response: Y-BOCS decrease > 35%	4.07 (±3.49)	In the brief DUI subgroup response rate was significantly increased and Y-BOCS score percentage changes higher after 48-week follow-up (*p* < 0.001). In a logistic regression analysis, a shorter DUI was predictive of a better response (*p* = 0.003). DUI was positively associated with DI but not with age of onset; this revealed that longer DUI indicates a longer clinical course.	YES
		Patients were treated with selective serotonin reuptake inhibitors or venlafaxine for 48 weeks in open-label conditions.					

BABS = Brown Assessment of Beliefs Scale; BAI = Beck Anxiety Inventory; BDI = Beck Depression Inventory; CGI scores = Clinical Global Impression Scale; DI = Duration of Illness; DSM-IV-TR = Diagnostic and Statistical Manual of Mental Disorders-IV-Text Revision; DUI = Duration of Untreated Illness; DY-BOCS = Dimensional Yale–Brown Obsessive–Compulsive Scale; ERP = Exposure and Response Prevention; GAF = Global Assessment of Functioning Scale; GCBT = Group cognitive–behavioural therapy; HAM-A = Hamilton Anxiety Rating Scale; HAM-D = Hamilton Depression Rating Scale; HDRS = Hamilton Depression Rating Scale; OCD = Obsessive–Compulsive Disorder; SCID I = Structured Clinical Interview for DSM IV Axis I Disorders; SCID II = Structured Clinical Interview for DSM IV Axis II; SSRI = Serotonin Reuptake Inhibitors; Y-BOCS = Yale–Brown Obsessive–Compulsive Scale; SD= Standard Deviation; OR= Odds Ratio. * DUI originally reported in months.

## Supplementary Materials

The following supporting information can be downloaded at: https://www.mdpi.com/article/10.3390/jpm13101453/s1, Table S1: Risk of Bias assessment in non randomized clinical studies.
