# Supplementary material for: Duration of Untreated Illness in Patients with Obsessive–Compulsive Disorder and Its Impact on Long-Term Outcome: A Systematic Review
Source: J Pers Med. 2023 Sep 29;13(10):1453. doi: 10.3390/jpm13101453 (PMC10608019; doi:10.3390/jpm13101453)
Supplement: Supplementary file 1 [file jpm-13-01453-s001.zip › jpm-2637133-supplementary.pdf]

Table S1. Risk of Bias assessment in non randomized clinical studies.

Authors (year of publication)	Type of study	Pre-intervention domains			At-intervention domain	Post-intervention domains			Overall Risk of Bias
		Confounding bias	*Selection bias*	*Information bias*	*Confounding bias*	*Selection bias*	*Information bias*	*Reporting bias*	
**Dell'Osso et al. (2010) [30]**	Longitudinal study	Moderate	Moderate	Low	Moderate	Low	Low	Moderate	Moderate
**Jakubovski et al. (2013) [31]**	Longitudinal study	High	Moderate	Low	High	Moderate	High	High	High
**Dell'Osso et al. (2015) [32]**	Cross-sectional study	High	Low	Moderate	Low	Low	Moderate	Moderate	High
**Poyraz et al. (2015) [33]**	Cross-sectional study	Moderate	Low	Low	Moderate	Low	Low	Low	Moderate
**Dell'Osso et al. (2017) [34]**	Cross-sectional study	Moderate	Low	Low	Low	Low	Moderate	Low	Moderate
**Albert et al. (2019) [17]**	Retrospective study	Moderate	Low	Low	Low	Low	Moderate	Moderate	Moderate
**Perris et al. (2021) [35]**	Longitudinal study	Low	Low	Low	Low	Low	Low	Low	Low
**Zheng et al. (2021) [36]**	Longitudinal study	Moderate	Low	Low	Moderate	Low	Moderate	Low	Moderate
